# Melanoma patients with additional primary cancers: a single-center retrospective analysis

**DOI:** 10.18632/oncotarget.26931

**Published:** 2019-05-21

**Authors:** Florentia Dimitriou, Joanna Mangana, Alessandra Curioni-Fontecedro, Markus Rechsteiner, Patrick Turko, Ralph P. Braun, Reinhard Dummer, Phil F. Cheng

**Affiliations:** ^1^ Department of Dermatology, University Hospital of Zurich, Zurich, Switzerland; ^2^ Department of Hematology and Oncology, Division of Oncology, University Hospital of Zurich, Zurich, Switzerland; ^3^ Department of Pathology and Molecular Pathology, University Hospital of Zurich, Zurich, Switzerland; ^4^ Faculty of Medicine, University of Zurich, Zurich, Switzerland

**Keywords:** melanoma, additional primary tumors, multiple primary tumors, BRAF V600E mutation, papillary thyroid cancer

## Abstract

**Background:** Recent progress in the diagnosis and treatment of primary and metastatic cutaneous melanoma (CM) has led to a significant increase in the patients` expectancy of life. The development of additional primary tumors (APT) other than CM represents an important survival issue.

**Results:** Of a total of 1764 CM patients, 80 (4.5%) patients developed APT. For tumors diagnosed after CM, there was a 2.7 fold excess risk for APT compared to the swiss german population. A significantly increased risk was noted for female breast (SIR, 2.46), male larynx (SIR, 76.92), male multiple myeloma (SIR, 11.2), male oesophagus (SIR, 10.8) and thyroid on males (SIR, 58.8) and females (SIR, 38.1). All thyroid cancer cases had a common papillary histological subtype and a high rate of BRAFV600E mutation. Melanoma was the primary cause of death in the vast majority of patients.

**Methods:** We used the cancer registry from the Comprehensive Cancer Center Zurich (CCCZ) and retrospectively analyzed patients with CM and APT between 2008 and 2018. We calculated the risk of APT compared to the swiss german population using the standardized incidence ratio (SIR).

**Conclusions:** Patients with CM have an increased risk for hematologic and solid APT. Long-term follow-up is indicated.

## INTRODUCTION

Cancer is a genetic disease, caused by the accumulation of genetic mutations that eventually transform a normal cell into a tumor cell [1]. It is of great concern for industrial countries due to the increasing incidence rates of the most common forms of cancer, mostly due to the lengthening of human lifespan and population aging [2, 3]. Cutaneous melanoma (CM) is, when not diagnosed early, an aggressive skin cancer derived from the melanocytes [4]. Although it represents approximately 5% of all cutaneous malignancies, it is considered one of the most lethal form of all skin cancers. Recent advances in understanding the molecular mechanisms and the underlying pathogenesis of the disease has led to significant improvements in the diagnosis and treatment and subsequently to an increase of the melanoma survival rates [5]. Due to variable environmental factors resulting in DNA damage, melanoma is associated with a high burden of somatic mutations [6, 7]. The identification of frequent mutations in the MAPK pathway has led to the development of targeted inhibitors, such as BRAF inhibitors (BRAFi) and MEK inhibitors (MEKi), with improved response and overall survival (OS) [8]. On the other hand, melanoma is an immunogenic cancer, conferring sensitivity to immunotherapeutic antibodies, which augment the cell-mediated immunity, such as checkpoint inhibitors against cytotoxic T-lymphocyte-associated antigen 4 (CTLA-4) or programmed cell-death protein 1 (PD-1) [9]. Due to this remarkable progress in metastatic melanoma treatment, today, the proportion of patients alive at 24 months in first-line setting is 62.9% with anti-PD1 plus anti-CTLA4 and 53.5% with BRAFi and MEKi [10].

Considering the tremendous improvement in the OS, it is also important to focus on survival issues and consequences, such as the development of long-term toxicities and most importantly new, independent tumors of different primaries other than melanoma. SEER-database analyses have previously shown that melanoma patients are prone to the development of other primary tumors of different histological type [11] and vice versa; other malignancies have been related to a secondary melanoma development [12–17]. Since the diagnosis and treatment complexity of the patients with additional primary tumors (APT) increases, we retrospectively analyzed melanoma patients with primary tumors of histological type other than melanoma in our melanoma-reference center. We aimed to describe the distribution of these tumors analog to melanoma diagnosis, underline the complexity of these patients and emphasize the importance of long-term follow-up in patients diagnosed with cancer.

## RESULTS

### Study population

**Figure 1:**
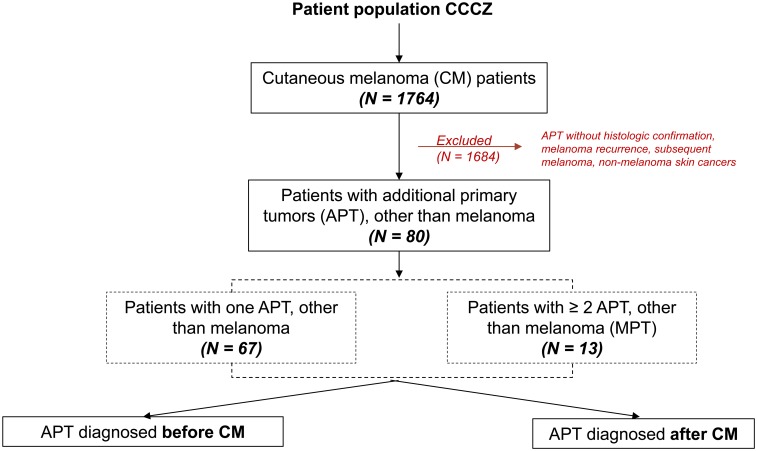
Patient population, CCCZ: Comprehensive Cancer Center Zurich.

There was a total of one thousand seven hundred and sixty four (*n* = 1764) patients diagnosed with melanoma between 2008 and 2018 with a cut-off of June 2018. Of the 1764 melanoma patients, eighty (4.5%) patients were diagnosed with an APT, from which thirteen (16.25%) patients developed multiple (> three) separate cancer types of different primary (MPT) (Figure 1). The median patient age at melanoma diagnosis was 70 years (33-90 years) and the majority of the patients were males (65%). Thirty (37.5%) patients had a family history of cancer, with same cancer in first- or second-degree relatives in 8.8%. 60% of the patients diagnosed with an APT had metastatic melanoma of which 26.7% were metastatic to the brain. Since mutational analysis does not belong to the standard tests for patients with non-metastatic melanoma in our institution and 32.4% of the patients were stage III-IV, mutational status was known in 57% of the patients. 26.6% of these patients were *BRAF V600* mutated and 16.5% *NRAS*. As of June 2018 69.6% of the patients with APT were alive. Of the 30.4% deceased, metastatic melanoma was the primary cause of death in the vast majority of patients (79.2%), followed by breast cancer (8.3%). Other causes of death were multiple myeloma (4.2%), pancreas cancer (4.2%) and lung cancer (4.2%). Patients’ characteristics, demographics and features of melanoma are listed in Table 1.

**Table 1:** Basic demographic characteristics of study population

		Overall
N		80
**Age at first diagnosis (median (range))**		70 (33–80)
**Age group (%)**	18,34	1 (1.2)
	35,49	7 (8.8)
	50,69	38 (47.5)
	70,90	34 (42.5)
**Family history (%)**	First degree relative same cancer type	4 (5.0)
	First degree relative different cancer type	21 (26.2)
	Second degree relative same cancer type	3 (3.8)
	Second degree relative different cancer type	2 (2.5)
	No family history of cancer	36 (45.0)
	Unknown	14 (17.5)
**Smoking (%)**	No	57 (71.2)
	1–9py	4 (5.0)
	10–19py	4 (5.0)
	20–29py	5 (6.2)
	30py+	7 (8.8)
	Unknown	3 (3.8)
**Race (%)**	Asian	1 (1.2)
	White	79 (98.8)
**Sex (%)**	Female	28 (35.0)
	Male	52 (65.0)
**Localisation of CM (%)**	Head and neck	17 (21.2)
	Trunk	27 (33.8)
	Upper extremities	9 (11.2)
	Lower extremities	13 (16.2)
	Acral	6 (7.5)
	Mucosal and uveal	3 (3.8)
	unknown	5 (6.2)
**Type of CM(%)**	SSM	17 (21.2)
	NM	25 (31.2)
	LMM	6 (7.5)
	ALM	5 (6.2)
	Unknown primary	6 (7.5)
	Mucosal or Uveal	1 (1.2)
	Unclassified	15 (18.8)
	Unknown	5 (6.2)
**Breslow (%)**	0–1 mm	10 (12.7)
	1.01–2 mm	29 (36.7)
	2.01–4 mm	6 (7.6)
	>4 mm	23 (29.1)
	Unknown	11 (13.9)
**Ulceration (%)**	No	35 (44.3)
	Yes	22 (27.8)
	Unknown	22 (27.8)
**Mutation Status (%)**	BRAF	21 (26.6)
	NRAS	13 (16.5)
	BRAF wild-type	3 (3.8)
	BRAF and NRAS wild-type	5 (6.3)
	cKIT	2 (2.5)
	MTOR	1 (1.3)
	Unknown	34 (43.0)
**AJCC Stage at first diagnosis (%)**	Stage I or II	54 (67.5)
	Stage III	21 (26.2)
	Stage IV	5 (6.2)
**Metastatic Melanoma (%)**	No	32 (40.0)
	Yes	48 (60.0)
**CNS Metastasis (%)**	No	33 (73.3)
	Yes	12 (26.7)
**Status (%)**	Alive	55 (69.6)
	Deceased	24 (30.4)
**Death due to (%)**	Breast	2 (8.3)
	Lung	1 (4.2)
	Melanoma	19 (79.2)
	Multiple Myeloma	1 (4.2)
	Pancreas	1 (4.2)

Abbreviations: CM: cutaneous melanoma.

### Additional primary tumors (APT)

In all, we found 93 second primary cancers, accounting also for multiple cancers, diagnosed before and after the first melanoma diagnosis between January 2008 and June 2018. The most frequently observed APT was prostate cancer in males (24.7%) followed by breast cancer in females (15.1%). Other frequently observed cancers on the overall distribution were bladder cancer (7.5%), lung cancer (6.5%), thyroid cancer (6.5%), non-hodgkin lymphoma (5.4%) and leukemia (5.4%) (Table 2). No predominant leukemia type was found. There was diversity in the stage of first diagnosis of the APT with 31.2% in stage I, IA and IB and 16.2% in stage IV and IVA. Consequently, 44.1% of these APT received curative surgical resection only, 27.9% received curative resection combined either with radiotherapy (11.8%) or with systemic therapy (11.8%) or both (4.3%). Altogether, 30.2% of the patients in our cohort required a systemic therapy; 10.8% alone, 11.8% combined with operation, 1.1% with radiotherapy, 4.3% with both operation and radiotherapy and 2.12% with radiotherapy and bone marrow- or stem cell- transplantation. 6.5% of the APT were under follow up only. For information on the course of treatment see Table 2.

**Table 2:** Additional primary tumors` (APT) characteristics

		Both sexes	Female		Male	
		Overall	After primary melanoma	Before primary melanoma	After primary melanoma	Before primary melanoma
***N***		93	18	14	28	33
**APT type (%)**	Bladder	7 (7.5)	0 (0.0)	1 (7.1)	2 (7.1)	4 (12.1)
	Brain & Central Nerves	1 (1.1)	0 (0.0)	0 (0.0)	1 (3.6)	0 (0.0)
	Breast	14 (15.1)	8 (44.4)	5 (35.7)	0 (0.0)	1 (3.0)
	Cervix Uteri	1 (1.1)	0 (0.0)	1 (7.1)	0 (0.0)	0 (0.0)
	Colon, Rectum	3 (3.2)	1 (5.6)	1 (7.1)	1 (3.6)	0 (0.0)
	Eye	1 (1.1)	1 (5.6)	0 (0.0)	0 (0.0)	0 (0.0)
	Kidney	3 (3.2)	1 (5.6)	0 (0.0)	1 (3.6)	1 (3.0)
	Larynx	1 (1.1)	0 (0.0)	0 (0.0)	1 (3.6)	0 (0.0)
	Leukaemia	5 (5.4)	0 (0.0)	4 (28.6)	1 (3.6)	0 (0.0)
	Lung, Bronchus, Trachea	6 (6.5)	2 (11.1)	1 (7.1)	2 (7.1)	1 (3.0)
	Merkel	1 (1.1)	0 (0.0)	0 (0.0)	1 (3.6)	0 (0.0)
	Multiple Myeloma	3 (3.2)	0 (0.0)	0 (0.0)	2 (7.1)	1 (3.0)
	Nerve sheath	1 (1.1)	0 (0.0)	0 (0.0)	0 (0.0)	1 (3.0)
	Non Hodgkin Lymphoma	5 (5.4)	1 (5.6)	0 (0.0)	1 (3.6)	3 (9.1)
	Oesophagus	2 (2.2)	0 (0.0)	0 (0.0)	2 (7.1)	0 (0.0)
	Oral Cavity & Pharynx	3 (3.2)	0 (0.0)	0 (0.0)	1 (3.6)	2 (6.1)
	Pancreas	4 (4.3)	0 (0.0)	1 (7.1)	2 (7.1)	1 (3.0)
	Pleura	1 (1.1)	0 (0.0)	0 (0.0)	1 (3.6)	0 (0.0)
	Prostate	23 (24.7)	0 (0.0)	0 (0.0)	6 (21.4)	17 (51.5)
	Stomach	1 (1.1)	0 (0.0)	0 (0.0)	1 (3.6)	0 (0.0)
	Testis	1 (1.1)	0 (0.0)	0 (0.0)	0 (0.0)	1 (3.0)
	Thyroid	6 (6.5)	4 (22.2)	0 (0.0)	2 (7.1)	0 (0.0)
**Stage AJCC/Rai (%)**	I	20 (21.5)	5 (27.8)	2 (14.3)	7 (25.0)	6 (18.2)
	IA	5 (5.4)	2 (11.1)	2 (14.3)	0 (0.0)	1 (3.0)
	IB	4 (4.3)	2 (11.1)	0 (0.0)	2 (7.1)	0 (0.0)
	II	2 (2.2)	0 (0.0)	0 (0.0)	2 (7.1)	0 (0.0)
	IIA	8 (8.6)	2 (11.1)	1 (7.1)	1 (3.6)	4 (12.1)
	IIB	8 (8.6)	1 (5.6)	0 (0.0)	1 (3.6)	6 (18.2)
	III	6 (6.5)	0 (0.0)	1 (7.1)	2 (7.1)	3 (9.1)
	IIIA	3 (3.2)	0 (0.0)	1 (7.1)	1 (3.6)	1 (3.0)
	IIIB	2 (2.2)	1 (5.6)	1 (7.1)	0 (0.0)	0 (0.0)
	in situ	2 (2.2)	0 (0.0)	1 (7.1)	0 (0.0)	1 (3.0)
	IV	13 (14.0)	3 (16.7)	1 (7.1)	5 (17.9)	4 (12.1)
	IVA	2 (2.2)	0 (0.0)	0 (0.0)	1 (3.6)	1 (3.0)
	Unknown	18 (19.4)	2 (11.1)	4 (28.6)	6 (21.4)	6 (18.2)
**Treatment type (%)**	None	6 (6.5)	1 (5.6)	0 (0.0)	4 (14.3)	1 (3.0)
	Operation	41 (44.1)	7 (38.9)	3 (21.4)	12 (42.9)	19 (57.6)
	Operation and Radiotherapy	11 (11.8)	2 (11.1)	3 (21.4)	3 (10.7)	3 (9.1)
	Operation and Radiotherapy and Systemic therapy	4 (4.3)	3 (16.7)	0 (0.0)	1 (3.6)	0 (0.0)
	Operation and Systemic therapy	11 (11.8)	3 (16.7)	4 (28.6)	3 (10.7)	1 (3.0)
	Radiotherapy	7 (7.5)	2 (11.1)	0 (0.0)	3 (10.7)	2 (6.1)
	Radiotherapy and Systemic therapy	1 (1.1)	0 (0.0)	0 (0.0)	0 (0.0)	1 (3.0)
	Radiotherapy and Systemic therapy and Bone marrow transplantation	1 (1.1)	0 (0.0)	1 (7.1)	0 (0.0)	0 (0.0)
	Systemic therapy	10 (10.8)	0 (0.0)	2 (14.3)	2 (7.1)	6 (18.2)
	Systemic therapy and Stem cell transplantation	1 (1.1)	0 (0.0)	1 (7.1)	0 (0.0)	0 (0.0)
**Course (%)**	CR	40 (43.0)	5 (27.8)	6 (42.9)	10 (35.7)	19 (57.6)
	PD	12 (12.9)	3 (16.7)	2 (14.3)	6 (21.4)	1 (3.0)
	PR	1 (1.1)	1 (5.6)	0 (0.0)	0 (0.0)	0 (0.0)
	SD	39 (41.9)	9 (50.0)	6 (42.9)	12 (42.9)	12 (36.4)
	Unknown	1 (1.1)	0 (0.0)	0 (0.0)	0 (0.0)	1 (3.0)

Abbreviations: CR: complete response, PR: partial response, PD: progressive disease, SD: stable disease.

### Comparison of APT diagnosed before and after melanoma

**Figure 2:**
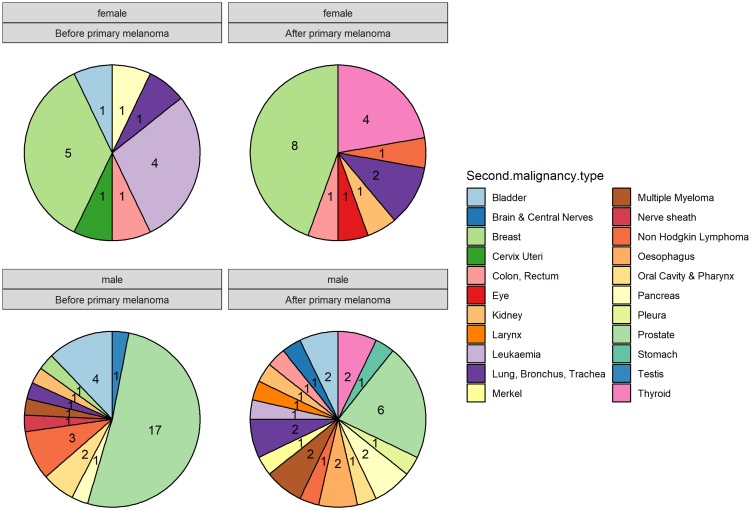
Comparison of additional primary tumors (APT) diagnosed before and after melanoma according to sex.

**Figure 3:**
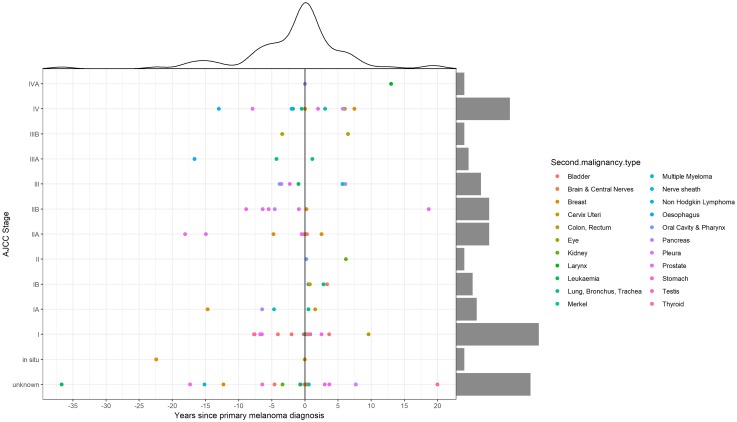
Time and staging distribution of additional primary tumors (APT) according to cutaneous melanoma (CM) diagnosis.

We compared the APT diagnosed before (50.5%) and after (49.5%) the first melanoma diagnosis and analyzed for differences in site of occurrence according to sex (Figure 2). Breast cancer in females and prostate cancer in males remain the most frequently observed APT before and after the melanoma diagnosis, with a frequency of 12.8% and 17.4% respectively for breast cancer and 36.2% and 13% for prostate cancer (Table 2). Bladder cancer in males occurs with a frequency of 8.5% before CM and 4.3% after CM. In females, the second most frequent APT is leukemia (8.5%) before CM with no cases after CM and thyroid cancer (8.6%) after CM with no cases before CM. In our patient cohort, all thyroid cancer cases were observed after the first melanoma diagnosis in both sexes. On the contrary, the vast majority of hematologic malignancies (leukemia in females and non-hodgkin lymphoma in males) seem to occur before the melanoma diagnosis. Median time to APT diagnosis after CM was 15.91 months (range 0–241.16) and median time to CM after APT diagnosis was 55.37 months (0.30–443.17) (Figure 3). On APT diagnosed before CM, 23.4% were stage I and 12.7% stage IV, whereas on APT diagnosed after 39.1% were stage I and 19.6% stage IV (Figure 3). DCR was achieved in 91.5% on APT before CM and 80.5% after CM.

For APT diagnosed after CM, we additionally analyzed the patterns of diagnosis, concluding that 28 out of 50 APT were diagnosed at melanoma follow-up, including PET/CT and CT imaging, as well as clinical examination.

### Standardized incidence ratio (SIR) analysis

**Figure 4:**
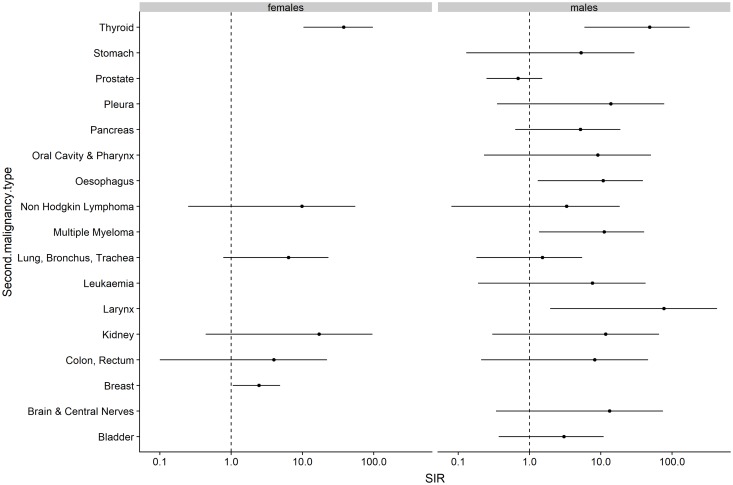
Risk of additional primary tumors (APT) diagnosed after cutaneous melanoma (CM) in the study population compared to the expected cancer incidence rates in the German Swiss population. Abbreviations: SIR: Standardized Incidence Ratios with 95% Confidence Interval.

Although we had a small patient cohort, we calculated the expected number of APT after first melanoma diagnosis assuming that cancer risk in the cohort was similar to that observed in the German Swiss population (Figure 4). We found a few cancers with elevated incidence; breast cancers in females [SIR 2.46 (1.06–4.86)], larynx in males [SIR 76.92 (1.01–427.9)], multiple myeloma in males [SIR 11.2 (1.36–40.6)], oesophagus in males [SIR 10.8 (1.31–39.1)] and thyroid cancers in both females [SIR 38.1 (10.38–97.54)] and males [SIR 48.8 (5.91–176.2). Overall, we found the SIR for our melanoma cohort to be 2.71 (1.96–3.63). Interestingly, prostate cancer in males showed a lower incidence after CM diagnosis [SIR 0.69 (0.25–1.51)].

### Cutaneous melanoma and papillary thyroid cancer

Based on our findings that all thyroid cancer cases (6.5%) occurred after the first melanoma diagnosis and due their common papillary pathological type (PTC), although our small patient cohort, we aimed to assess the rate of *BRAF V600E* mutation in this patient cohort. We therefore performed a real-time quantitative PCR procedure (Idylla) of all available tumor tissues. Six patients with both CM and PTC were tested, of which 4 were found to be positive for *BRAF V600E* mutation in melanoma, 6 for *BRAF V600E* in PTC and 4 in both.

## METHODS

### Patient selection and data collection

The cancer registry of the Comprehensive Cancer Center Zurich (CCCZ) is a melanoma reference database with centralized data and quality management, for skin cancer. CCCZ was queried for cutaneous melanoma (CM) patients with additional primary tumors (APT) between the years of 2008 and 2018, with a closing date of June 2018 and a minimum follow up time of 6 months. Patients with non-melanoma skin cancers (NMSC) besides Merkel Cell Carcinoma (MCC), melanoma recurrence, subsequent second or third melanomas and benign tumors were excluded. Since APT in patients with metastatic disease are difficult to distinguish from melanoma metastases, we only included APT with a histologic confirmation. The tumors were classified according to the American Joint Committee on Cancer (AJCC) 7th edition. Geographic, histopathologic and treatment data after diagnosis of metastatic disease were retrospectively collected for all patients. Response evaluation to the systemic treatment was according to the radiologic RECIST 1.1 criteria. Patients` records were also searched for risk factors, including family history, smoke, age, gender and race. In order for the reported variables not to contribute in more than one category, for patients with a first and second degree relative with cancer, only the first degree relative was included. In order to describe the distribution of APT with respect to melanoma diagnosis, we labelled the patients into two groups; APT before and after CM. Multiple primary tumors (MPT) were defined as two or more separate neoplasms of different primary, other than melanoma. Follow-up time was calculated from the day of resection of the CM to the date of last follow-up, including last visit or date of death, or June 2018, whichever occurred first.

For APT occurring after CM, the standardized incidence ratios (SIRs) were calculated by dividing the observed numbers of cancer by the expected ones. The observed numbers of cancers and person-years at risk were calculated by gender, 5-year age group and the time since the diagnosis of CM. The expected numbers of cancer were obtained by multiplying the stratum-specific numbers of person-years by the corresponding cancer incidence rates in German Swiss population in Switzerland extracted from the Nationales Institut für Krebsepidemiologie und -registrierung (NICER) database. Exact 95% confidence intervals (CIs) were defined when the numbers of observed cases followed a Poisson distribution. All analyses were conducted using statistical language R version 3.5.

Written informed consent for retrospective analysis of melanoma patients in our registry was previously approved by local ethics committee (KEK-ZH 2014-0193).

### DISCUSSION AND CONCLUSIONS

On our retrospective analysis, there is an overall incidence of 4.5% of an APT before and after CM diagnosis, among of which 16.25% attributed to MPT. Based on our analysis, we show that patients who were previously diagnosed with cutaneous melanoma (CM) have approximately a 2.7 fold increased risk of an APT compared to the general Swiss German population. These results are consistent with previous international findings reporting a significantly elevated risk for specific subsequent primary cancers other than melanoma in melanoma survivors [11], although the relatively greater overall risk of 28% described in the cited study. Albeit, a direct comparison to these data could be misleading, taking into consideration the differences in the population size, follow-up duration as well as inclusion criteria. Since patients with CM have an increased risk of second primary CM, subsequent melanomas were excluded from our analysis [11, 18–20]. In our patient cohort, female breast cancer, male larynx cancer, male multiple myeloma, male oesophagus cancer and thyroid cancer in both sexes had statistically increased incidence after the first melanoma diagnosis, compared to the normal population. The reasons for this increased risk seem to be multifactorial and could be contributed to increased medical surveillance and follow-up after CM diagnosis, as well as other independent, non-modifiable risk factors, such as the presence of an inherited genetic predisposition and advanced age [21, 22], and behavioral, modifiable risk factors, such as UV-exposure [23] and immunosuppression [24–26], especially for hematologic tumors. A mutual association between CM and female breast cancer has been reported in both genetic and population-based retrospective studies [27], suggesting a higher prevalence of both cancers for mutations of high-risk genes, such as *BRCA2* and CM [28] and *CDKN2A* and breast cancer [29, 30]. Unorthodoxly, the incidence of prostate cancer in our population seems to decrease after CM diagnosis, although this result should be cautiously interpreted, due to the relatively low number of this event in our patient cohort. The latter may be of particular interest, since several studies have connected CM and prostate cancer with common risk factors responsible for the pathogenesis of both cancer types, such as the UV exposure, with subsequently increased risk of prostate cancer diagnosis after melanoma diagnosis [12, 16, 31–34]. On the other hand, the role of androgens and oestrogens in the melanoma development have been controversially discussed [35–38]; altogether, the assumption of an additional biological or hormonal relationship between CM, prostate cancer and breast cancer remains unclear.

Interestingly, the probability of a new thyroid cancer diagnosis seems to increase after CM diagnosis in both sexes, which is in accordance of previous reported results [11]. Besides, the controversial relationship between CM and thyroid cancer has been confirmed from previous retrospective reviews, suggesting that patients with papillary thyroid cancer (PTC) are at significantly increased risk of CM and vice versa [39]. The precise etiology for this correlation is unclear; however, the thyroid cancer cases observed in our patient cohort had a common papillary histological subtype and a high rate of *BRAFV600E* mutational background, which was observed in both CM and PTC, thus unravelling a possible common genetic component of these cancers. This is of great importance, considering that several types of cancer are a result of mutations in critical genes [40]. Still, although every individual tumor is genetically distinct, the pathways affected in different tumors may overlap [6]. Approximately 50% of the CM cases harbor an activating mutation in the *BRAF* oncogene [41], whereas *BRAF V600E* mutations have been also described in approximately 44% of patients with papillary thyroid cancer (PTC) [42]. These results may indicate a common genetic pathway in the pathogenesis of these cancers.

Our dataset showed that in patients with APT or MPT, metastatic melanoma still remains the most common cause of death. Yet, a large proportion of the APT are usually diagnosed on early stages with high DCR rates. There was also a significant difference among the median time of the diagnosis for APT before and after CM ((55.37 months (0.30–443.17) versus 15.91 months (0–241.16)), which can be attributed to the patterns of care after CM diagnosis with regular screening during follow-up. Indeed, 28 out of 50 APT diagnosed after CM were detected at melanoma follow-up, including PET/CT and CT imaging or clinical examination. The Swiss guidelines recommend an increased vigilance of follow-up in the first 10 years, with surveillance every 3 to 6 months the first 5 years and every 12 months for the next 5 years for the stage I-II melanoma [43]. Patients with a previous diagnosis of CM are more likely to have more regular and attentive health controls, which may increase the possibility of detecting APT and thus in an early stage. Still, there was a slight difference between stage IV APT diagnosed before (12.7%) and after (19.6%) CM, although a possible explanation for this finding is unclear.

The main strength of our study is that the CCCZ registry provides a high accuracy in tumors registering with long and minimum loss of follow-up. Such patients are usually excluded from clinical trial protocols, thus only few prospective data are available. Our study emphasizes the complexity of patients with unrelated APT and the importance of increased follow-up for the prompt detection of such survival issues. Further research is required to unravel the burden and causative relationship of APT and primary tumors, as well as their socioeconomic impact on the survival population.

#### Ethics statement

Written informed consent for retrospective analysis of melanoma patients in our registry was previously approved by local ethics committee (KEK-ZH 2014-0193).
